# Evaluation of Î³-oryzanol content and composition from the grains of pigmented rice-germplasms by LC-DAD-ESI/MS

**DOI:** 10.1186/1756-0500-6-149

**Published:** 2013-04-15

**Authors:** Heon Woong Kim, Jung Bong Kim, Poovan Shanmugavelan, Se Na Kim, Young Sook Cho, Haeng Ran Kim, Jeong-Tae Lee, Weon-Tai Jeon, Dong Jin Lee

**Affiliations:** 1 Department of Agro-food Resources, National Academy of Agricultural Science, Rural Development Administration, Suwon, 441-883, Republic of Korea; 2 National Institute of Crop Science, RDA, Suwon, 441-857, Republic of Korea; 3 Department of Crop Science and Biotechnology, Dankook University, Cheonan, 330-714, Republic of Korea

## Abstract

**Background:**

Rice is the staple food and one of the worldâ€™s three major grain crops. Rice contains more than 100 bioactive substances including phytic acid, isovitexin, Î³-oryzanol, phytosterols, octacosanol, squalene, Î³-aminobutyric acid (GABA), tocopherol, tocotrienol derivatives, etc. Out of them, Î³-oryzanol is known to have important biological profile such as anti-oxidants, inhibitor of cholesterol oxidation, reduce serum cholesterol levels in animals, effective in the treatment of inflammatory diseases, inhibit tumor growth, reduce blood pressure and promotes food storage stability when used as a food additive, etc. Hence in the present investigation, we aimed to evaluate the content and composition of Î³-oryzanol from pigmented rice germplasms using a liquid chromatography with diode array detection and electrospray ionization-mass spectrometry (LC-DAD-ESI/MS).

**Findings:**

In the present study, 33 exotic pigmented rice accessions (red, white and purple) have been evaluated. Among them, the contents of Î³-oryzanol varied from 3.5 to 21.0Âmg/100Âg with a mean of 11.2Âmg/100Âg. A total of ten components of Î³-oryzanol including âˆ†^7^-stigmastenyl ferulate were identified of which, cycloartenyl ferulate, 24-methylenecycloartanyl ferulate, campesteryl ferulate and sitosteryl ferulate were identified as the major components. The mean proportions of steryl ferulates were in the descending order of 24-methylenecycloartanyl ferulate > cycloartenyl ferulate > campesteryl ferulate > sitosteryl ferulate > âˆ†^7^-campestenyl ferulate > campestanyl ferulate > sitostanyl ferulate > âˆ†^7^-stigmastenyl ferulate > stigamsteryl ferulate > âˆ†^7^-sitostenyl ferulate. Almost 11 accessions (33%) showed higher content than the control rice *Chucheongbyeo* and higher proportions ranged from 10 to 15Âmg/100Âg. Interestingly, the red rice accession Liberian Coll. B11/B-11 (21.0Âmg/100Âg) showed higher content Î³-oryzanol than control rice *Jeokjinjubyeo* (19.1Âmg/100Âg) and the purple rice accession Padi Adong Dumarat, Mardi No.4376 (20.3Âmg/100Âg) showed a similar content with control rice *Heugjinjubyeo* (21.4Âmg/100Âg).

**Conclusions:**

Most of analyzed rice accessions were found to possess higher contents of Î³-oryzanol than the control rice, *Chucheongbyeo.* In particular, the red accessions showed highest content than the white and purple accessions. The content and composition of Î³-oryzanol in 33 exotic pigmented rice accessions have been evaluated and compared significantly by the present investigation.

## Background

Rice is the staple food for more than half the worldâ€™s population and a valuable food resource as one of the worldâ€™s three major grain crops. Particularly in Asia, it is a primary food source for most of the countries which canâ€™t be replaced by any other crops. However, the rice consumption is showing a considerable downward trend every year as a consequence of the westernization, diversification of dietary patterns and deprived consideration of the nutritional excellence of rice-based diets. Consequently, there is an essential need to upsurge the rice consumption and to solve the economic/social problems of farming communities by develop the rice varieties with more improved nutritional quality and functionality [1-3].

Generally, rice contains more than 100 bioactive substances mainly in its bran layer including phytic acid, isovitexin, Î³-oryzanol, phytosterols, octacosanol, squalene, Î³-aminobutyric acid (GABA), tocopherol, tocotrienol derivatives, and some new substances have also been identified and isolated from the rice accessions [4-6]. Out of them, Î³-oryzanol (mixture of ferulic acid esters of triterpene alcohols and sterols) and lipid-soluble substances (tocopherol and tocotrienol) are known to be the most powerful anti-oxidants [7]. Remarkably, the total content of Î³-oryzanol composition in rice bran is 13â€“20 times higher than that of tocopherols and tocotrienols. Hence, they are known to have ten times higher anti-oxidant activity than tocopherol derivatives [8,9]. In addition, Î³-oryzanol is a potent inhibitor of cholesterol oxidation [10-12], reduce serum cholesterol levels in animals [13-15], effective in the treatment of inflammatory diseases [16,17], effective in anxiety neurosis and menopausal disorders [18-20], inhibit tumor growth [16,21], helps to lower blood pressure [22] and promotes food storage stability when used as a food additives [23,24]. On the other hand, ferulic acid esters are found in the seeds of grain crops such as rice, wild rice, corn, wheat, rye and barley. Among them, rice has been reported to contain most ferulic acid esters [25-29] which are well-known for their biological importance. In particular, pigmented rice has the functionality of pigments such as anthocyanin, procyanidin, catechin, catecholtannin, etc., and bioactive substances such as Î³-oryzanol, phytosterols, octacosanol, etc., in the bran layer and thus provides excellent anti-oxidant activity compared to other common rice varieties [30-32].

At first, Î³-Oryzanol was found in rice bran oil in 1954 [33] and it was thought that it consisted of a single component. Later, it was found to be a mixture of different components through a high performance liquid chromatographic analysis (HPLC). However, the number of identified individual components has varied depending on the chromatographic approaches. Diack et al. [34] attempted to separate Î³-oryzanol into two fractions using a normal-phase HPLC on a silica column, but their approach failed to isolate and identify the individual components. In contrast, when reverse-phase HPLC was used, Norton et al. [35] and Miller et al. (2003) [36] succeeded in isolating the five different components and Evershed et al. [25] and Rogers et al. [37] succeeded in isolating the six different components. In view of this, recently a reverse-phase HPLC technique has successfully isolated and identified the ten different phytosteryl ferulates of Î³-oryzanol including cycloartenyl ferulate, 24-methylenecycloartanyl ferulate and campesteryl ferulate as major components by Xu et al. [38]. Owing to fewer studies on the content and composition of Î³-oryzanol in genetic resources and earlier chromatographic analysis involves limitations associated with low peak resolution, long analysis time and large sample size to detect minor components in brown rice, we were tempted to attain an analysis which suggests to irradiate the above limitations and involves a large-scale screening in the evaluation of genetic resources.

Thus, in connection with the above issues and in continuation of our earlier report on the evaluation of anthocyanins in colored potatoes by LC-DAD-ESI-MS [39], the present investigation intends to describe the evaluation of Î³-oryzanol content and composition in the seeds of pigmented-rice genetic resources using a liquid chromatography associated with diode array detection and electrospray ionization-mass spectrometry [LC-DAD-ESI/MS].

## Findings

FigureÂ1 shows the HPLC chromatograms of standard Î³-oryzanol components and extracts from the seeds of control rice varieties, *Chucheongbyeo* (white rice) and *Heugjinjubyeo* (red rice). In the present study, a total of 10 components were isolated of which, cycloartenyl ferulate, 24-methylenecycloartanyl ferulate, campesteryl ferulate and sitosteryl ferulate were identified as the major components. Interestingly, herein analysis time was shortened to 40Âminutes compared with previous report of 60Âminutes and the peak retention time ranged between 15â€“30Âminutes [38,40,41].

**Figure 1:**
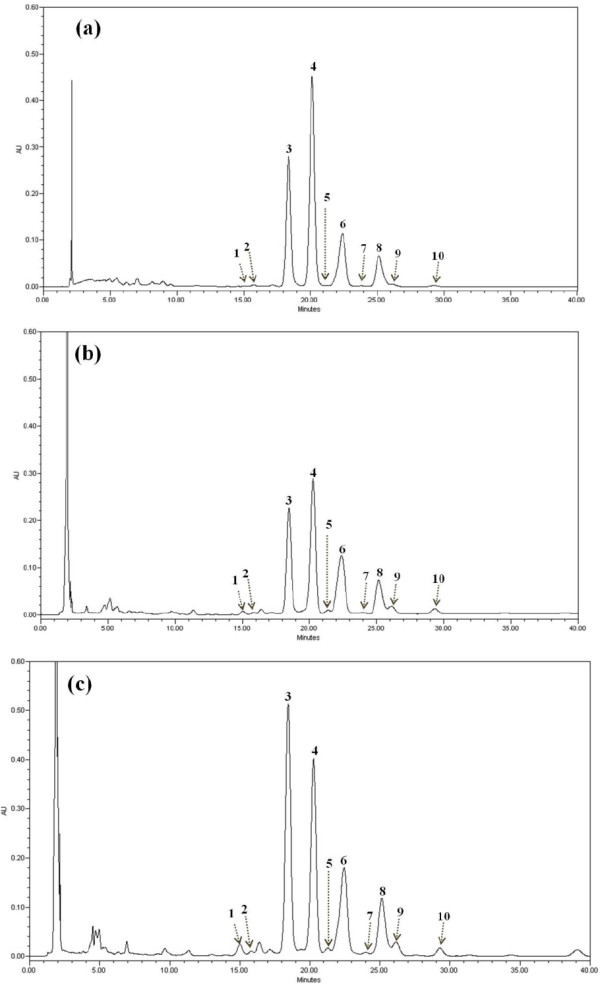
**HPLC chromatograms of Î³-oryzanol extracted from grains of the rice germplasm (1: âˆ†**^**7**^**-stigmastenyl ferulate, 2: stigamsteryl ferulate, 3: cycloartenyl ferulate, 4: 24-methylenecycloartanyl ferulate, 5: âˆ†**^**7**^**-campestenyl ferulate, 6: campesteryl ferulate, 7: âˆ†**^**7**^**-sitostenyl ferulate, 8: sitosteryl ferulate, 9: campestanyl ferulate, 10: sitostanyl ferulate).**

In particular, eight pigmented rice accessions have relatively higher content of Î³-oryzanol with more than 15Âmg/100Âg. Padi Adong Dumarat Mardi No.4376, a purple rice accession showed a similar Î³-oryzanol content of 20.3Âmg/100Âg to the control rice *Heugjinjubyeo* (21.4Âmg/100Âg), and Î³- Liberian Coll. B11/B-11, a red rice accession showed higher content of 21.0Âmg/100Âg than the control rice *Jeokjinjubyeo* (19.1Âmg/100Âg). The eight pigmented rice accessions showed higher Î³-oryzanol content than control-group in the order of 4.5-6 times higher than *Chucheongbyeo* (3.5Âmg/100Âg), 2.5-3.5 times higher than *Baekjinjubyeo* (6.1Âmg/100Âg), and 1.8-2.5 times higher than *Keunnunbyeo* (8.8Âmg/100Âg) (TableÂ1). The chemical structure of each isolated component was derived on the basis of mass spectral information from GC-MS and LC-MS analysis [29,39]. When analysis was conducted in negative-ionization mode on a single quadrupole MS equipped with an ESI source, the forehead portion from the whole structure of each isolated component was found to appear as ferulic acid moiety (*m/z* 177, 178, 193) based on fragment pattern of methyl group (CH_3_; *m/z* 15) (FigureÂ2).

**Table 1:** Î³-Oryzanol contents and compositions of steryl and triterpene alcohol ferulates in 33 pigmented rice germplasms collected from IRRI

**Accessions**	**Steryl and triterpene alcohol ferulates, proportions in total Î³-oryzanol (%)**												**Î³-oryzanol content (mg/100Âg hulled rice)**
	**Origin**	**Seed coat color**	**âˆ†**^**7**^**-stigma stenyl ferulate**	**Stigma steryl ferulate**	**24-Methylene cycloartanyl ferulate**	**Cyclo artenyl ferulate**	**âˆ†**^**7**^**-campe stenyl ferulate**	**Campe Steryl ferulate**	**âˆ†**^**7**^**-sito stenyl ferulate**	**Sito Steryl ferulate**	**Campe stanyl ferulate**	**Sito stanyl ferulate**	
Hill Padi Mardi No. 4337	Malaysia	Purple	3.2	1.4	26.6	31.8	10.9	13.5	0.3	8.6	2.3	1.4	17.9
Padi Adong Dumarat Mardi No. 4376	Malaysia	Purple	2.2	3.0	23.7	30.5	10.7	17.6	0.2	9.9	1.3	0.9	20.3
Tadong 1	Malaysia	Purple	1.2	0.5	29.7	28.7	8.5	17.1	0.3	11.7	1.3	1.0	8.8
Pulut Tindal ARC 818/57/85/222	Malaysia	Purple	1.4	1.3	29.0	28.0	6.5	16.3	0.7	9.6	4.5	2.8	12.3
Padi Pagalon Mardi No. 4370	Malaysia	Red	1.3	0.5	27.0	33.6	12.0	11.9	0.2	8.1	2.8	2.6	5.5
Paditarab. Arab Mardi No. 4400	Malaysia	Red	1.5	0.8	25.5	29.1	9.8	20.0	0.4	12.1	0.3	0.6	10.2
Akuramboda/5 BAD86	Sri Lanka	Red	1.6	1.7	13.8	36.5	18.2	17.2	0.3	7.3	2.4	1.1	15.1
Balawee/19-005	Sri Lanka	Red	1.3	1.1	22.3	41.2	5.2	16.8	0.1	8.4	2.1	1.5	17.8
Mada Al/19-001	Sri Lanka	Red	0.8	0.5	16.9	42.0	6.9	18.5	0.2	9.3	3.0	2.0	10.6
Kalu Galpa Wee	Sri Lanka	Red	1.0	0.7	22.5	35.3	5.1	18.5	N.D. ^a^	9.4	4.9	2.6	8.8
Matta Thatwel/17/84/100	Sri Lanka	Red	0.5	0.2	20.2	37.1	14.5	8.7	1.0	8.5	5.1	4.3	7.9
260 FAO ACC. 29. 793	Liberia	Red	1.0	0.5	23.4	30.4	11.7	17.2	0.3	9.8	3.6	2.0	4.3
545 FAO ACC. 29. 832	Liberia	Red	1.2	1.1	38.3	23.2	3.2	16.1	0.2	10.8	3.6	2.4	6.1
Quakor/AGI-34	Liberia	Red	1.2	1.0	38.4	24.7	2.9	16.3	0.2	10.7	2.9	1.8	12.3
Sayllebon/3-150	Liberia	Red	0.9	1.4	32.7	27.8	3.5	17.5	0.3	10.3	3.6	2.2	7.8
Sayllebon/3-203	Liberia	Red	1.4	1.1	36.6	25.8	3.1	18.5	0.1	12.0	0.5	1.0	9.1
Liberian Coll. B11/B-11	Liberia	Red	1.5	0.9	34.9	25.4	3.6	16.7	0.1	10.8	3.6	2.5	21.0
Liberian Coll. D4-84/D4-84	Liberia	Red	1.4	1.2	34.7	26.9	4.0	15.5	0.5	11.4	2.8	1.8	12.2
Kwandwo Amoa	Ghana	Red	2.9	1.2	24.5	31.1	11.3	16.0	0.3	8.9	2.5	1.5	16.7
Gbotokole Tos13129/YS137	Ghana	Red	1.3	0.4	28.2	30.0	9.1	15.5	0.2	10.7	2.8	1.8	1.9
Tinsibe/YS188	Ghana	Red	1.2	0.4	25.0	29.0	9.5	17.9	0.1	12.0	2.7	2.2	11.3
Beselen/YS678	Ghana	Red	1.1	0.5	28.6	28.7	8.5	14.8	0.2	10.6	4.3	2.8	14.1
IS16 TOS7542/IS16	Ivory Coast	Red	1.2	0.8	41.6	24.9	3.3	16.1	0.2	11.0	0.3	0.8	17.5
Sahima TOS9552/IS186	Ivory Coast	Red	1.1	0.4	25.0	30.9	10.9	15.8	0.2	10.3	3.1	2.2	10.0
Joboi TOS6984	Sierra Leone	Red	1.2	0.6	24.4	28.8	8.3	16.5	0.3	13.9	3.1	3.0	14.0
Koni TOS6922	Sierra Leone	Red	1.3	0.6	25.9	30.5	8.4	16.9	0.2	10.9	2.9	2.4	7.3
Gbondobai TOS7490/SL 11-288	Sierra Leone	Red	0.9	0.8	35.4	29.1	7.3	14.2	0.4	7.9	2.5	1.6	12.0
Gbonelobai TOS7501/SL 11-384	Sierra Leone	Red	1.1	0.6	36.3	28.3	6.7	12.2	0.2	9.8	2.6	2.3	5.7
ARC 13256	India	Red	1.1	0.7	21.1	36.1	12.6	12.7	0.6	8.6	3.7	2.9	3.5
Mamadou Salif TOS11927/SS628	Senegal	Red	1.6	1.2	32.8	28.6	4.6	16.5	0.3	8.5	3.8	2.1	15.3
Bidjoco TOS10530/BS153	Guinea Bissau	Red	0.8	0.5	24.2	30.3	11.0	20.9	0.4	9.5	1.5	0.9	14.7
Boro 1	Bangladesh	Red	3.1	2.5	10.5	35.7	17.6	18.0	0.2	7.2	3.7	1.5	13.0
Nanton53/PI245061	Taiwan	Purple	1.2	1.0	29.1	32.0	9.2	14.1	0.2	8.7	2.9	1.7	4.0
		Mean	1.4	0.9	27.5	30.7	8.4	16.1	0.3	9.9	2.8	1.9	11.2
		S.D. ^b^	0.6	0.6	7.2	4.5	4.0	2.4	0.2	1.5	1.2	0.8	5.0

^a^*N.D.* : Not detected.

^b^*S.D.* : Standard deviation.

**Figure 2:**
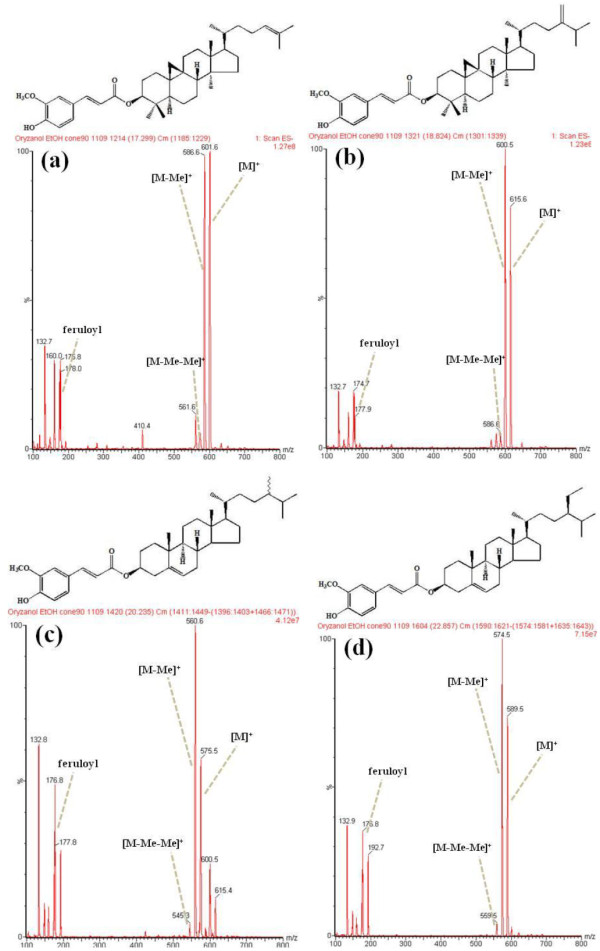
Structures and MS spectra of major Î³-oryzanol components isolated from grains of Korean white rice cultivar, Chucheongbyeo.

A reverse-phase HPLC analysis of 5 purple and 28 red rice accessions as pigmented-rice genetic resources showed the total contents of Î³-oryzanol ranged from 3.5Âmg/100Âg to 21.0Âmg/100Âg with a mean of 11.2Âmg/100Âg. This is less than half of the total contents of 30Âmg/100Âg [42] and 40Âmg/100Âg [41] in earlier studies. This difference appears due to the differences between rice varieties or the number of peaks separated in the HPLC chromatogram and the area of each peak. The Î³-oryzanol content was 10â€“15Âmg/100Âg in 11 rice accessions (33%) and less than 5Âmg/100Âg in four accessions (12%). Most of the rice accessions showed a higher Î³-oryzanol content than the control rice, chucheongbyeo (3.5Âmg/100Âg) (TableÂ2).

**Table 2:** Î³-Oryzanol contents and compositions of steryl and triterpene alcohol ferulates in 7 Korean rice samples (control rice varieties)

**Accessions**	**Steryl and triterpene alcohol ferulates, proportions in total Î³-oryzanol (%)**												**Î³-oryzanol content (mg/100Âg hulled rice)**
	**Origin**	**Seed coat color**	**âˆ†**^**7**^**-stigma stenyl ferulate**	**Stigma steryl ferulate**	**24-Methylene cycloartanyl ferulate**	**Cyclo artenyl ferulate**	**âˆ†**^**7**^**-campe stenyl ferulate**	**Campe Steryl ferulate**	**âˆ†**^**7**^**-sito stenyl ferulate**	**Sito Steryl ferulate**	**Campe stanyl ferulate**	**Sito stanyl ferulate**	
Chucheongbyeo	Korea	White	2.5	3.8	27.7	35.6	0.8	16.3	0.2	9.5	2.3	1.3	3.5
Kunnunbyeo	Korea	White	5.1	2.7	23.2	32.5	13.0	10.3	1.2	8.8	1.8	1.6	8.8
Baekjinjubyeo	Korea	White	1.1	0.7	30.5	33.6	6.3	13.0	0.1	9.4	2.8	2.6	6.1
Hanyangjo	Korea	Red	1.1	0.6	20.8	35.8	12.9	15.4	0.1	7.2	4.5	1.8	14.3
Chosundo	Korea	Red	1.5	1.6	20.7	30.4	10.1	19.7	0.7	11.9	2.1	1.3	13.6
Jeokjinjubyeo	Korea	Red	1.5	1.2	37.2	28.6	4.3	16.2	0.3	10.3	0.2	0.4	19.1
Heugjinjubyeo	Korea	Purple	0.9	2.6	36.8	29.9	0.5	15.9	0.3	8.3	3.0	1.8	21.4
		Mean	1.9	1.9	28.1	32.3	6.8	15.3	0.4	9.4	2.4	1.5	12.4
		S.D. ^a^	1.5	1.2	7.0	2.8	5.3	3.0	0.4	1.5	1.3	0.7	6.6

^a^*S.D.* : Standard deviation.

FigureÂ3 describes the mean proportions of individual components of Î³-oryzanol extracted from 33 pigmented rice accessions. The highest proportion was found in the order of 24-methylenecycloartanyl ferulate (23.17-41.96%, mean = 30.7%), cycloartenyl ferulate (10.47-41.59%, mean = 27.5%), campesteryl ferulate (8.70-20.90%, mean = 16.1%), sitosteryl ferulate (7.22-13.85%, mean = 9.9%), âˆ†^7^-campestenyl ferulate (2.87-18.19%, mean = 8.4%), campestanyl ferulate (0.25-5.06%, mean = 2.8%), sitostanyl ferulate (0.62-4.34%, mean = 1.9%), âˆ†^7^-stigmastenyl ferulate (0.50-3.20%, mean = 1.4%), stigamsteryl ferulate (0.23-3.00%, mean = 0.9%), and âˆ†^7^-sitostenyl ferulate (0.09-0.66%, mean 0.3%). The major Î³-oryzanol components were found as cycloartenyl ferulate, 24-methylenecycloartanyl ferulate, campesteryl ferulate and sitosteryl ferulate in control rice varieties *Heugjinjubyeo* and *Jeokjinjubyeo*. But, cycloartenyl ferulate was found as the highest proportion of total Î³-oryzanol components in IS16 TOS7542/IS16, *Heugjinjubyeo*, Tadong 1, Pulut Tindal ARC 818/57/85/222.

**Figure 3:**
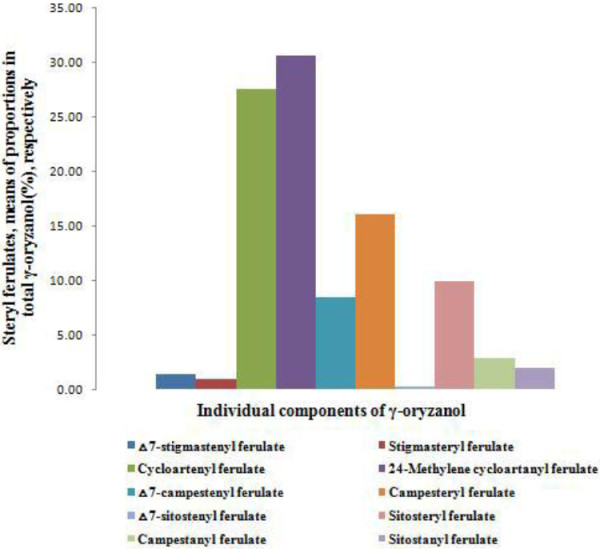
Comparison of the means proportions of total individual components (%) isolated from the grains of pigmented rice germplasms.

FigureÂ4 describes the scores plotting chart of principal components 1 and 2 of the PLS-DA results obtained from the data set by Î³-oryzanol profiling on the red rice germplasms and thus we could identify overall patterns, variations and cluster formations at a glance. The plotting of PLS-DA scores for correlations between the content and composition of Î³-oryzanol in 40 rice accessions collected from Africa and Asia region. It found no specific cluster formation in terms of seed coat color (white, red or purple), but 31 red-rice accessions showed cluster formations depending on the place of origin (East Africa and Southeast Asia) (FigureÂ4a). Principal component 1 (PC 1) and principal component 2 (PC 2) represented the variations of 31.5% and 24.5%, respectively with a total of 56%. The VIP value indicating a high importance (â‰¥ 1) in cluster formation which was highest in component 4 (24-methylenecycloartanyl ferulate, 1.57) followed by component 3 (cycloartenyl ferulate, 1.29), component 8 (sitosteryl ferulate, 1.14), and component 5 (â–³^7^-campestenyl ferulate, 1.07) (FigureÂ4b) and thus, components 3, 4 and 8 were major and component 5 was minor component in their Î³-oryzanol composition. For the strains belonging to six countries of Northwest coast of Africa (Ghana, Liberia, Sierra Leone, the Ivory Coast, Guinea Bissau and Senegal), cycloartenyl ferulate was found to higher than 24-methylenecycloartanyl ferulate in the proportion of individual components, whereas the accessions from four Southeast Asian countries (Sri Lanka, Malaysia, Bangladesh and India) were found to have higher proportions of 24-methylenecycloartanyl ferulates than cycloartenyl ferulates. Consequently, the main components, cycloartenyl ferulate and 24-methylenecycloartanyl ferulate were considered to have a major role in the formation of clusters by the place of origin. Further, upon the evaluation of genetic resources from a variety of regions, other individual components are also thought to play an important role as biomarkers.

**Figure 4:**
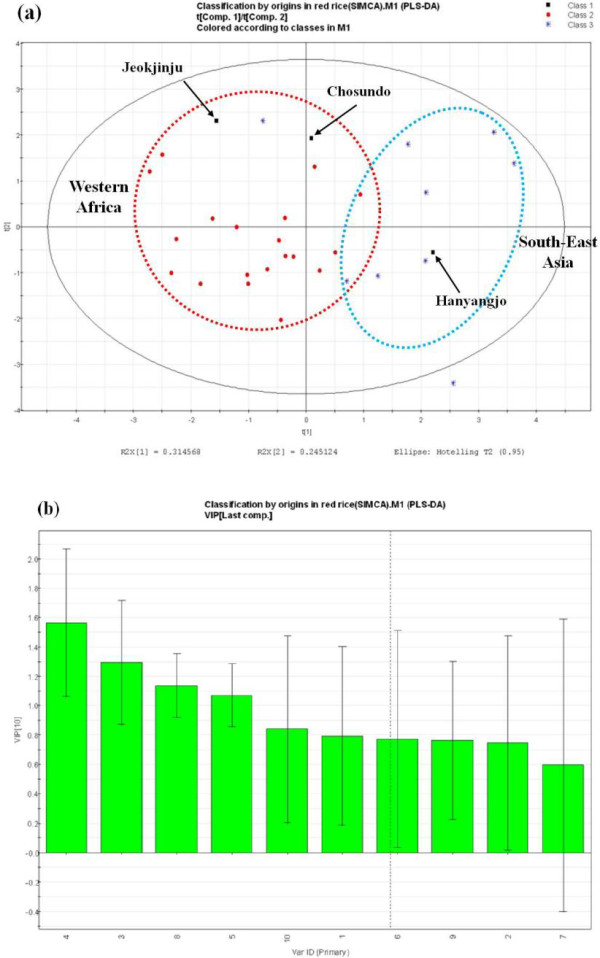
**Scores plotting chart of principal components 1 and 2 of PLS-DA results obtained from the data set by Î³-oryzanol profiling on the red rice germplasms.** (**a**) classifications by origin of the all samples (red circle: Western Africa; blue triangle: South-East Asia; black box: Korean red rice cultivar), (**b**) influence of variable for this classifications (the value of variable importance in the prediction, VIP).

## Conclusions

In the present investigation, the analysis of contents and compositions of Î³-oryzanol from 33 exotic pigmented rice accessions and 7 Korean rice varieties using LC-DAD-ESI/MS has been accomplished. As a result, the contents of Î³-oryzanol varied from 3.5 to 21.0Âmg/100Âg with a mean of 11.2Âmg/100Âg. A total of ten components of Î³-oryzanol including âˆ†^7^-stigmastenyl ferulate were identified of which, cycloartenyl ferulate, 24-methylenecycloartanyl ferulate, campesteryl ferulate and sitosteryl ferulate were identified as the major components. The mean proportions of steryl ferulates were in the descending order of 24-methylenecycloartanyl ferulate > cycloartenyl ferulate > campesteryl ferulate > sitosteryl ferulate > âˆ†^7^-campestenyl ferulate > campestanyl ferulate > sitostanyl ferulate > âˆ†^7^-stigmastenyl ferulate > stigamsteryl ferulate > âˆ†^7^-sitostenyl ferulate. Almost 11 accessions (33%) showed higher content than the control rice *Chucheongbyeo* and higher proportions ranged from 10 to 15Âmg/100Âg. Interestingly, the red rice accession Liberian Coll. B11/B-11 (21.0Âmg/100Âg) showed higher content Î³-oryzanol than control rice *Jeokjinjubyeo* (19.1Âmg/100Âg) and the purple rice accession Padi Adong Dumarat, Mardi No.4376 (20.3Âmg/100Âg) showed a similar content with control rice *Heugjinjubyeo* (21.4Âmg/100Âg). Most of analyzed rice accessions were found to possess higher contents of Î³-oryzanol than the control rice, *Chucheongbyeo.* In genral, the red accessions showed highest content than the white and purple accessions.

## Materials and methods

### Materials

In this study, 33 pigmented rice accessions (5 purple and 28 red accessions) and 7 Korean rice varieties (3 white, 3 red and 1 purple rice varieties) obtained from International Rice Research Institute (IRRI) were used for analysis. Hulled rices (whole grain) were used in this study.

### Instrumentation and reagents

The instruments were used for the pretreatment process included a refrigerated multi-purpose centrifuge (Hanil Science Industrial Co. Ltd., Korea), and an ultrasonic bath (Daihan Scientific Co. Ltd., Korea). Î³-Oryzanol (Wako, Japan) was used as an external standard. The HPLC reagents were methanol, dichloromethane, acetic acid and acetonitrile from Sigma (St. Louis, MO).

### Extraction of Î³-Oryzanol

A 5Âg powdered sample in a conical tube (50ÂmL) was centrifuged (3000Ârpm, 10Âmin, 4Â°C) following extraction with 40ÂmL of dichloromethane-methanol (2:1, v/v) for 30Âminutes at 30Â°C in an ultrasonic shaking water bath. One millilitre of the supernatant solution was collected from the centrifuged sample. A Sep-Pak C_18_ cartridge was flushed with 10ÂmL of dichloromethane followed by the addition of 10ÂmL of methanol for activation. After loading 1ÂmL of supernatant (Î³-Oryzanol extract) and lÂmL of standard solution (Î³-Oryzanol, 1000Âppm), the cartridge was washed with 10ÂmL of dichloromethane and eluted with 1ÂmL of methanol. The Î³-Oryzanol filtrate was eluted and concentrated using N_2_ gas, and then re-dissolved in dichloromethane-methanol (2:1, v/v) prior to analysis with LC-DAD-ESI/MS.

### Quantitative and qualitative analysis of Î³-Oryzanol by LC-DAD-ESI/MS

The quantitative and qualitative analysis Î³-Oryzanol in the grains of red and purple rice accessions (whole grain) were analyzed using a Micromass ZQ MS (Waters Co., Milford, MA) and an Alliance e2695 HPLC system (Waters Co.) equipped with a 2998 photodiode array detector (PDA). In addition, YMC PACK ODS-AM reversed-phase column (4.6 x 250Ânm I.D., 5ÂÎ¼m; YMC Co. Ltd, Japan) was used. The analysis was conducted at a flow rate of 1.4ÂmL/min in the detection wavelength of 250â€“400Ânm (a representative wavelength of 325Ânm) with the column heater temperature of 30Â°C. The mobile phases used were methanol: acetonitrile:dichloromethane:acetic acid (50:44:3:3, v/v/v/v) with isocratic elution for 40Âminute. The MS was analyzed in negative ionization mode using electrospray ionization (ESI) source. The MS parameters were a cone voltage of 90ÂV, source temperature of 100Â°C, desolvation temperature of 500Â°C, and a desolvation N_2_ gas flow of 480Âl/h. The range of molecular weights was *m/z*, 100â€“800 in full scan mode.

### Partial least squares discriminant analysis (PLS-DA)

The SIMCA P+ software (version: 11, Umetrics AB, Umea, Sweden) for multivariate data analysis was used to acquire PLS-DA for discriminate the analyzed rice varieties by arranging and normalizing all quantitative information obtained in this study.

## Competing interests

The authors declared that they have no competing interests.

## Authorsâ€™ contributions

**HWK** performed the experiments. **PS**, equally contributor who wrote the manuscript. **JBK**, corresponding author who guided & supervised the work. **SNK, YSC, HRK, LJT, JWT** and **DJL** participated in designing of the work. All authors read and approved the final version of the manuscript.
